# RETURN-TO-WORK AFTER ACETABULAR FRACTURES: THE IMPACT OF INJURY SEVERITY ON THE POST-REHABILITATION WORKING CAPACITY AND WORKLOAD

**DOI:** 10.2340/jrm-cc.v8.44156

**Published:** 2025-09-02

**Authors:** Anna L. SCHILTENWOLF, Tina HISTING, Maximilian M. MENGER, Christof K. AUDRETSCH, Florian LAUX, Markus A. KUEPER, Steven C. HERATH

**Affiliations:** 1 Department of Trauma and Reconstructive Surgery, Eberhard Karls University Tuebingen, BG Trauma Center Tuebingen, Tuebingen, Germany; 2 Health Care Center and Rehabilitation Clinic Swabian Alb, Bad Urach, Germany

**Keywords:** acetabular fracture, employment, polytrauma, return to work, rehabilitation, workload

## Abstract

**Objective:**

Acetabular fractures are among the most severe injuries in trauma surgery. In younger patients, they typically result from high-energy trauma and are often associated with polytrauma. Treatment complexity and rehabilitation outcomes are influenced by overall injury severity. This study aimed to evaluate return to work (RTW) after acetabular fracture in relation to the overall injury severity score (ISS).

**Design/subjects/patients:**

A retrospective study included 22 patients treated for acetabular fractures at a Level I Trauma Centre spanning a period from January 2009 – December 2020.

**Methods:**

Patients completed a questionnaire assessing work-related factors and workload before (PRE) and after (POST) trauma (median [MD] = 126.4 ± 46.4 months POST). Based on ISS, patients were categorized as < 16 = “no polytrauma” (*n* = 8) and ≥ 16 = “polytrauma” (*n* = 14).

**Results:**

The RTW rate was 75% in both groups. However, descriptively the median workload reduction was greater in the “polytrauma” group (–50%) compared to the “no polytrauma” group (–33.3%). A shift toward sedentary work was seen in both groups, more prominently in the “polytrauma” subgroup (+40%) compared to the “no polytrauma” group (+11.4%).

**Conclusion:**

Possible workload was reduced after acetabular fracture. Despite similar RTW rates, polytrauma patients descriptively returned to less physically demanding work. Thus, ISS significantly predicts the outcome of the rehabilitations process after acetabular fractures.

## LAY ABSTRACT

Acetabular fractures (fractures of the hip socket) are serious injuries, especially in younger people, often caused by high-energy accidents like car crashes. These injuries may occur alone or as part of multiple injuries, which can make recovery more difficult.

This study looked at 22 patients treated for acetabular fractures between 2009 and 2020. They answered questions about their job before and after the injury. Patients were divided into two groups based on injury severity: those with isolated fractures and those with polytrauma.

In both groups, 75% of patients were able to return to work (RTW). However, people with more severe injuries tended to return to less physically demanding jobs. Both groups showed a shift to more sedentary work, but this was more pronounced in the polytrauma group. Injury severity strongly influences recovery.

Acetabular fractures occur in a two-point age distribution (1). In younger individuals, acetabular fractures are most commonly caused by high-velocity trauma, whereas in older adults, they typically result from low-impact injuries combined with age-related bone loss (2). The aim of treating these fractures is, on the one hand, to reconstruct the hip socket, and on the other hand to restore independence and a satisfactory quality of life as well as reintegration into work (3–6).

After sustaining an acetabular fracture, between 60 and 90% of patients RTW (4, 5, 7). While the specific fracture classification appears to have little impact on RTW outcomes, several studies have identified factors such as age, pre-injury (PRE) employment status, and the presence of additional injuries as relevant predictors (5, 8).

However, these influencing variables all point toward an underlying factor: the overall severity of the injury. A more severe trauma with a higher Injury Severity Score (ISS) is likely to require longer hospitalization, more complex surgery, and extended rehabilitation – all of which may reduce the likelihood of RTW or regaining one’s previous workload capacity (9–11).

To date, no study has specifically examined whether overall injury severity, as measured by ISS, affects RTW or the post-injury (POST) workload in patients with acetabular fractures. The present study aims to address this gap.

## Methods

### Material and methods

Data for postal dispatch, socio-demographic data, and information on epidemiological classification with injury classifications and accident date were taken from the Level 1 Trauma Centre’s pelvic register database.

The acetabular fractures were classified according to Letournel’s classification (12). The individual injury severity was classified according to the AIS score and the overall injury severity according to the ISS score (13).

Surveys were conducted on work-related aspects, including employment status, job change, retirement, duration of incapacity to work (sick leave), employment relationship, working hours, salary changes, vocational retraining, and occupational reintegration.

The workload was determined and calculated based on the REFA criteria and the socio-medical standards applicable in Germany (14, 15). It was categorized into 4 levels: grade 1 = work with small physical strain, grade 2 = work with moderate physical strain grade 3 = work with hard physical strain, and grade 4 = work with most heavily physical strain. This classification considered factors such as exertion, weight to be lifted, duration, and frequency of the activity. Additionally, stressful postures and movement-related influences were considered (16–18).

### Patients collective

The present study included individuals who sustained an acetabular fracture and received treatment at a Level 1 Trauma Centre between January 2009 and December 2020. The required data were obtained from the database on pelvic injuries of this Level 1 Trauma Centre. The patients provided informed consent for participation and data collection/storage and were of working age (between 18 and 65 years of age) at the time of the trauma. The study was advised by the local ethical committee of the medical faculty under the project number 760/2021BO2. There were no objections to conducting the study.

### Data collection and analysis

The included study population was contacted in writing in October 2021 with an information letter, an informed consent form, and the questionnaire without blinding or stratification. The questionnaire contained exclusively retrospective self-assessments of the participants (patient reported outcome measures – PROMS). The questionnaire was returned alongside with the informed consent form. If the documents were incomplete, the patients were contacted by telephone and an attempt was made to complete the missing data. Subsequently, the questionnaires of the consented participants were evaluated pseudonymously. No physical examinations, imaging procedures or personal encounters were carried out as part of this study.

The analysis of the data was purely descriptive. No statistical tests were used.

## Results

### Participants

**Fig. 1:**
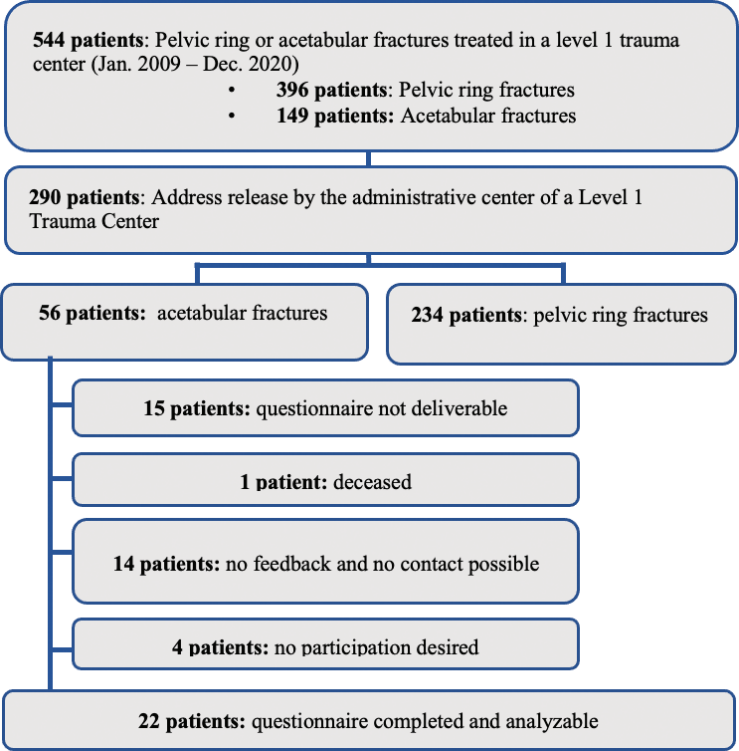
Flowchart of participants.

A total of 544 patients were recorded in the Level 1 Trauma Centre’s pelvic register for the specified period, 396 of them with pelvic ring fractures and 149 with acetabular fractures. The address of a total of 290 patients could be released by the administrative department of the Level 1 Trauma Centre, 56 of whom had an acetabular fracture and were included in the study. One patient was deceased at the time of contact. 15 patients could not be contacted by writing or telephone. Consequently, the questionnaire was successfully sent to 40 patients. Four of these patients declined to participate. The questionnaire could be delivered to 14 patients, but no response was received despite several attempts to contact them. Of the remaining 22 patients, fully completed questionnaires could be evaluated (response rate 22/40 = 55%) (Fig. 1).

Seven women (31.8%) and 15 men (68.2%) were included with an average age at the time of trauma of 45 ± 13.9 years (19–64). The average time between trauma and sending the questionnaire was 126.4 ± 46.4 months (50–184).

### Injury

There were 15 cases (68.2%) of elementary fracture patterns and 7 cases (31.8%) of combined fracture patterns (12). Based on the AIS score, 16 patients (72.7%) had an AIS score of 3, with 4 (18.2%) having a concomitant type B pelvic ring injury according to Tile’s classification (19). Six patients (27.3%) had an AIS score of 4 and sustained a concomitant pelvic ring fracture Tile type C. In total, 10/22 (45.5%) were affected by a concomitant pelvic ring fracture. According to the ISS, 14/22 (63.6%) suffered a polytrauma and 8/22 (36.4%) no polytrauma. In the overall collective, the mean ISS was 23.4 ± 15.0 (range 9–59). In the “no polytrauma” subgroup, the mean ISS was 9.8 ± 1.5 (range 9–13), while in the “polytrauma” subgroup it was 31.2 ± 13.5 (range 17–59).

The breakdown according to the subgroups “no polytrauma” and “polytrauma” can be found in Table I.

**Table I:** Characteristics of the subgroups

Characteristics	No polytrauma (ISS < 16)	Polytrauma (ISS≥16)
Age		
Median	49.5 years ± 9.9 (35–58)	43 years ± 15.5 (19–64)
Men : women	7 : 1	8 : 6
Body height		
Median	178 cm ± 5.9 (170–187)	171 cm ± 10 (155–186)
Body weight		
Median	82 kg ± 16.5 (60–111)	78 kg ± 14.8 (53–112)
AIS-Score (%)		
3	8/8 (100)	8/14 (57.1)
4	-	6/14 (42.9)
Additional pelvic ring fracture (%)		
Tile B	1/8 (12.5)	3/14 (21.4)
Tile C	-	6/14 (42.9)
Letournel/Judet (%)		
Anterior wall fracture	2/8 (25)	-
Anterior column fracture	4/8 (50)	2/14 (14.3)
Posterior column fracture	-	1/14 (7.1)
Transverse fracture	-	6/14 (42.8)
Transverse + posterior wall fracture	1/8 (12.5)	3/14 (21.4)
Both column fracture	1/8 (12.5)	2/14 (14.3)
Treatment pelvis (%)		
Conservative	3/8 (37.5)	1/14 (7)
One operation	2/8 (25)	3/14 (14.3)
Multiple operations	3/8 (37.5)	10/14 (57.1)
Rehabilitation (%)		
Yes	5/8 (62.5)	13/14 (92.9)
No	3/8 (37.5)	1/14 (7.1)
Duration outpatient rehab		
Median	60 days ± 11.5 (60–80)	21 days ± 35.8 (14–90)
Duration inpatient rehab		
Median	35 days ± 16.2 (21–60)	61.5 days ± 328.7 (21–1095)

ISS: Injury Severity Score; 2: AIS: Abbreviated Injury Score.

### Return to work

Of the total cohort, 9.1% (2/22) individuals were not working PRE, while 31.8% (7/22) were not working POST. Among the 20 individuals who were employed PRE, 15/22 returned to work POST, resulting in a RTW rate of 75%. This corresponds to a 22.7% decrease in employment (PRE: 20/22 [90.9%]; POST: 15/22 [68.2%]).

Among the 15 individuals who were employed POST, 6/15 (40%) had not sustained polytrauma, while 9/15 (60%) had experienced multiple traumatic injuries. In contrast, of the 7 individuals who did not RTW, 2/7 (28.6%) had not sustained polytrauma, while 5/7 (71.4%) were polytraumatized.

The work-related aspects of the 2 subgroups are detailed in Table II.

**Table II:** Work related aspects of the subgroups

Aspects	No polytrauma *n* = 8		Polytrauma *n* = 14	
	PRE	POST	PRE	POST
Employment (%)				
Yes	8/8 (100)	6/8 (75)	12/14 (85.7)	9/14 (64.3)
No	-	2/8 (25)	2/14 (14.3)	5/14 (35.7)
RTW rate (%)	6/8 (75)		9/12 (75)	
POST different employment than PRE (%)	2/8 (25)		4/14 (28.6)	
POST Retirement (%)				
Disability pension	2/8 (25)		4/14 (28.6)	
Old age pension	-		1/14 (7.1)	
Incapacity for work				
Median	136 ± 420.1 days (59–1147)		176 ± 565.0 days (95–1618)	
Employment relationship (%)				
Temporary employment	1/8 (12.5)	2/8 (25)	-	-
Permanent employment	4/8 (50)	3/8 (37.5)	8/14 (57.1)	7/14 (50)
Self-employment	2/8 (25)	1/8 (12.5)	2/14 (14.3)	1/14 (7.1)
Civil servant employment	-	-	1/14 (7.1)	1/14 (7.1)
Working hours (%)				
Part-time	-	-	1/14 (7.1)	-
Full-time	8/8 (100)	6/8 (75)	11/14 (78.6)	9/14 (64.3)
POST wage change (%)				
Higher salary	-		1/14 (7.1)	
Lower salary	1/8 (12.5)		-	
Same salary	4/8 (50)		8/14 (57.1)	
Not specified	1/8 (12.5)		-	
POST vocational retraining (%)				
Yes	2/8 (25)		2/14 (19.6)	
No	6/8 (75)		12/14 (85.7)	
POST occupational reintegration (%)				
No	3/8 (37.5)		6/14 (42.9)	
Yes, successfully completed	3/8 (37.5)		7/14 (50)	
Yes, bus cancelled	2/8 (25)		1/14 (7.1)	

PRE: pre-traumatic; POST: post-traumatic; RTW: return to work.

In the “no polytrauma” subgroup, the RTW rate was 75% (6/8), with a decrease in employment of 25% (PRE: 8/8 [100%]; POST: 6/8 [75%]). Additionally, 2/8 (25%) changed to another occupation POST. Vocational reintegration was undertaken by 5/8 (62.5%), with 3/8 (37.5%) successfully completing the program.

Similarly, the RTW rate in the “polytrauma” subgroup was also 75% (9/12). Employment decreased by 21.4% (PRE: 12/14 [85.7%]; POST: 9/14 [64.3%]). Of the post-traumatic working patients, 9/14 (64.3%) switched to another occupation. A vocational reintegration program was attended by 8/14 (57.1%), with 7/14 (50%) successfully completing it.

In the subgroup analysis, participants without polytrauma had an average length of stay that was almost 1/3 shorter than participants with polytrauma (“no polytrauma” MD = 4.5 months; “polytrauma” MD = 5.8 months). Due to the 40% shorter length of stay in inpatient rehabilitation in the “no polytrauma” subgroup, this result is not surprising.

### Workload

**Fig. 2:**
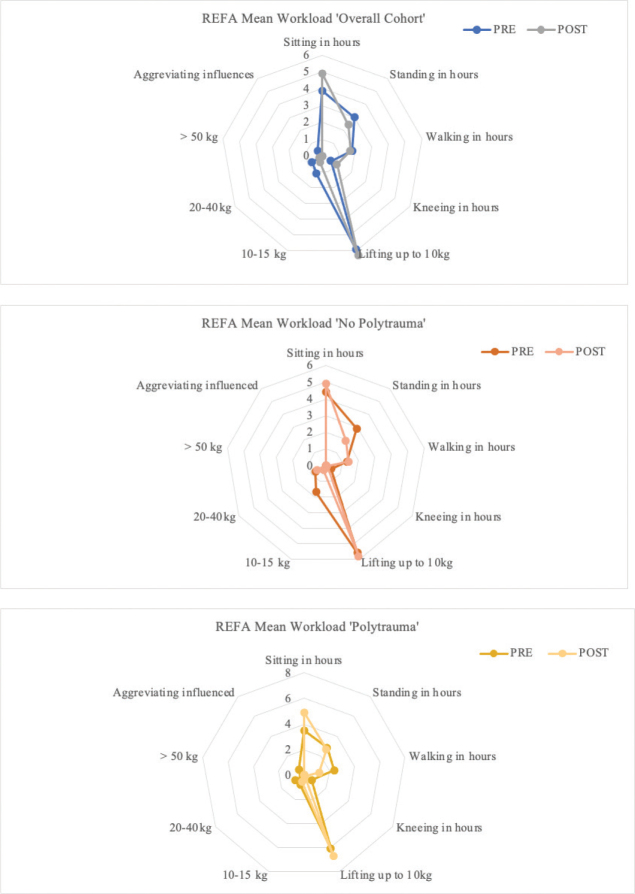
Workload according to REFA-classification Figure 2 shows the subitems used to calculate the workload according to the REFA-classification: In the participants without polytrauma, a decrease in the duration of time was recorded in every sub-item of the mean values, except for the categories “sitting,” “walking” and “lifting up to 10 kg”. The largest percentage decrease was seen in the category “lifting 10–15 kg” (–82.4%; M(PRE) = 1.7 h, min = 0, max = 10; M(POST) = 0.3 h, min = 0, max = 1). The largest percentage increase was found in the “sitting” category (+11.4%; M(PRE) = 4.4 h, min = 0.5, max = 8; M(POST) = 4.9 h, min = 0.5, max = 8). For participants with polytrauma, there was a decrease in the duration of time in every sub-item of the mean values, except for the categories “Sitting” and “lifting up to 10 kg.” The largest percentage decrease was seen in the categories “lifting 20–40 kg,” “lifting > 50 kg” and “Aggravating influences” (“lifting 20–40 kg”:–100%, M(PRE) = 0.8 h Min = 0, Max = 8, M(POST) = 0 h; “lifting > 50 kg”:–100%, M(PRE) = 0.1 h, Min = 0, Max = 0.5; M(POST) = 0 h; “aggravating influences”:–100%, M(PRE) = 0.6 h, Min = 0, Max = 4; M(POST) = 0 h). The largest percentage increase was found in the category “sitting” (+40%; M(PRE) = 3.5 h, Min = 0, Max = 8; M(POST) = 4.9 h, Min = 1, Max = 8).

**Fig. 3:**
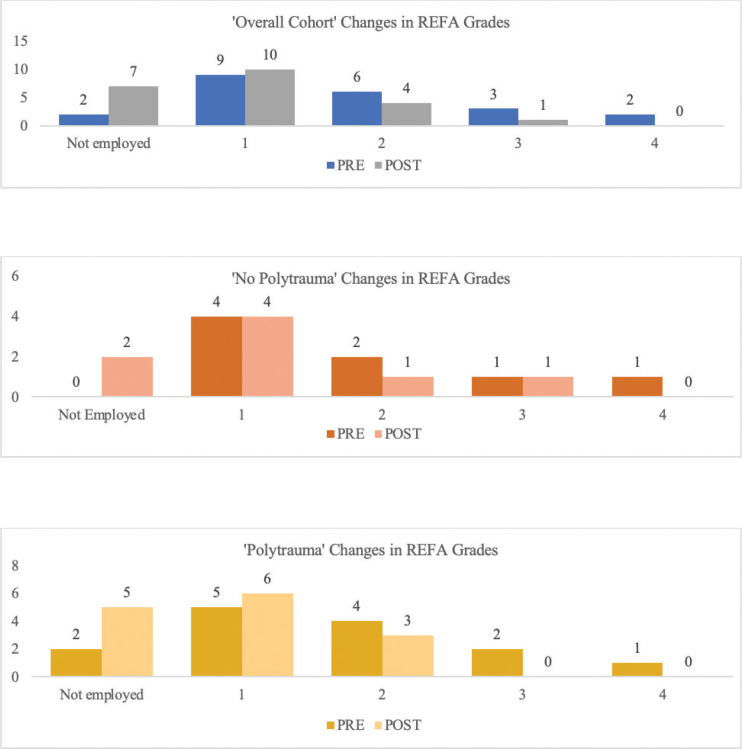
Changes in REFA grades Figure 3 shows the changes in the REFA grades (y-axis: number of samples; x-axis: answer in questionnaire PRE and POST)]: In the “no polytrauma” subgroup, workload descriptively reduced by 33.3% (PRE MD = 1.5 ± 1.1; POST MD = 1 ± 0.8). In the “polytrauma” subgroup, workload descriptively declined by 50% (PRE MD = 2 ± 1.0; POST MD = 1 ± 0.5).

In the overall cohort, the median of individual workload reduction (PRE – POST) was 0, while the mean reduction was 0.77, indicating a skewed distribution. Descriptively the median workload was reduced by 50% (PRE MD = 2 ± 1.0; POST MD = 1 ± 0.63). A closer analysis (Figs. 2 and 3) revealed that 59.1% of the patients maintained their workload PRE, while 40.91% experienced a reduction. Notably, no patients increased their workload following to the trauma.

In the “no polytrauma” subgroup, the median individual workload reduction was 0.5, with a mean of 0.72. Descriptively, the median of workload decreased by 33.3% (PRE MD = 1.5 ± 1.1; POST MD = 1 ± 0.8).

In the “polytrauma” subgroup, the workload reduction was 0, with a mean of 0.79, showing that while some patients did experience reduced workload, a majority maintained their previous level. Descriptively, the median workload was reduced by 50% (PRE MD = 2 ± 1.0; POST MD = 1 ± 0.5).

The corresponding sub-items and the calculation of workload are described in Table III and illustrated in Figs. 2 and 3.

**Table III:** Workload according to the REFA classification

Items of REFA classification	No polytrauma		Polytrauma	
	PRE	POST	PRE	POST
Mean subitems in hours				
Sitting	4.4 ± 3.1 (0.8–8)	4.9 ± 3.8 (0.5–8)	3.5 ± 3.1 (0.8)	4.9 ± 2.9 (1–8)
Standing	2.9 ± 2.3 (0–8)	1.9 ± 2.3 (0–6.5)	2.8 ± 2.0 (0.6)	2.7 ± 2 (0–6)
Walking	1.3 ± 1.0 (0–3)	1.4 ± 1.1 (0–3)	2.4 ± 1.9 (0–7)	1.2 ± 1.2 (0–3)
Kneeing	0.4 ± 0.7 (0–2)	0.1 ± 0.2 (0–0.5)	0.7 ± 1.1 (0–4)	0.1 ± 0.4 (0–0.5)
Lifting up to 10 kg	5.6 ± 3.2 (0–8)	5.8 ± 3.1 (1–8)	6.1 ± 2.5 (0–8)	6.7 ± 1.7 (4–8)
10–15 kg	1.7 ± 3.4 (0–10)	0.3 ± 0.4 (0–1)	0.8 ± 1.1 (0–3)	0.5 ± 0.9 (0–2)
20–40 kg	0.7 ± 1.8 (0–5)	0.6 ± 1.2 (0–3)	0.8 ± 2.3 (0–8)	-
> 50 kg	-	-	0.1 ± 1.1 (0–0.5)	-
Aggravating influences	-	-	0.6 ± 1.3 (0–4)	-
Workload (%)				
REFA grade 1	4/8 (50)	4/8 (50)	5/14 (14.3)	6/14 (42.9)
REFA grade 2	2/8 (25)	1/8 (12.5)	4/14 (28.6)	3/14 (21.4)
REFA grade 3	1/8 (12.5)	1/8 (12.5)	2/14 (14.3)	-
REFA grade 4	1/8 (12.5)	-	1/14 (7.1)	-

PRE: pre-traumatic; POST: post-traumatic; REFA: Reichsausschluss für Arbeitszeitermittlung.

### Gender difference

Within the total cohort (15 male, 7 female), all male individuals were working PRE (15/15; 100%), whereas two of the female individuals were not working PRE (2/7; 28.57%).

POST, four males did not RTW (4/15; 26.67%), resulting in a RTW rate of 73.33%. The median workload among males was descriptively reduced by 50% (PRE MD = 2 ± 1.13; POST MD = 1 ± 0.67). The median individual workload reduction was 1, with a mean reduction of 1. A total of 8 out of 15 males (53.33%) reported a reduced workload from PRE to POST.

Among the 5 female who were working PRE, 1 did not RTW (1/5; 20%), corresponding a RTW rate of 80%. The median workload was descriptively decreased by 25% (PRE MD = 2 ± 0,.8; POST MD = 1.5 ± 0.58). The median individual workload reduction was 0, with a mean of 0.29. Only 1 of 7 females (14.29%) experienced a reduction in workload PRE to POST.

In the “no polytrauma” subgroup (seven male, one female), two male individuals did not RTW (2/7; 28.57%), resulting in a male RTW rate of 71.43%. The sole female returned to work (1/1; 100%). Among males, median workload was descriptively decreased by 50% (PRE MD = 2 ± 1.15; POST MD = 1 ± 0.89), with a median workload individual reduction of 1 (mean = 0.86). The female workload remained unchanged (PRE and POST MD = 1 ± 0).

In the “polytrauma” subgroup (eight male, six female), two males did not RTW (2/7; 25%), leading to a RTW rate of 75%. Among females, two were not working PRE (2/6; 33.33%). Of the remaining four, one did not RTW (1/4; 25%), yielding a female RTW rate of 75%. Male median workload was descriptively reduced by 33.33% (PRE MD = 1.5 ± 1.20; POST MD = 1 ± 0.41) with a median individual workload reduction of 0.5 and a mean of 1.13.

Female workload did not decrease (PRE MD = 2 ± 0.5, POST MD = 2 ± 0.58), with a median individual reduction of 0 and a mean of 0.82.

## DISCUSSION

### Return to work

Descriptively, the present study shows that 15/22 (68.2%) of the total cohort did RTW, while 7/22 (31.8%) were unable to do so. Of the 20 individuals who were working PRE, 15 returned to work (15/20; 75%).

Monteleone et al. conducted a retrospective survey of 66 patients with surgically treated acetabular fractures. Among other things, they were asked about RTW according to the “Workplace Activity Limitation Survey” after a follow-up period of 65.1 months (20) with 64.6% of the individuals working PRE returning to work. Interestingly, the percentage of patients working PRE is lower when compared to the present study (75%). This discrepancy could be attributed to a lower percentage of pre-traumatic workers (73.8%) and a higher mean age of 53.4 years in the cohort study of Monteleone et al. (21).

As Nusser et al. identified in their retrospective study with 92 patients suffering from acetabular fractures, PRE employment status and age are the most important predictors of RTW (8).

The present results of the subgroup “no polytrauma” are similar to the results of Ng et al.. Ng et al. retrospectively examined 30 patients with surgically treated acetabular fractures regarding RTW after a follow-up period of 21.5 months. In the studied cohort, the mean ISS was 8.9, indicating the absence of polytrauma, with an observed RTW rate of 80.8%. However, according to Ng et al. more patients returned to the same occupation as PRE (18/21; 85.7%) when compared to the results of the present study (4/6; 66.7%). Notably, the average time interval between trauma and RTW in the study of Ng et al. (8.3 months) was longer than the average time interval determined in the present study (4.5 months). This may explain the higher reintegration into the pre-traumatic occupation, although this aspect was not recorded by Ng et al. (6).

Of the patients in the “no polytrauma” subgroup in this study, 62.5% took part in vocational rehabilitation, but only 37.5% of patients completed this successfully. Vocational rehabilitation has been shown to result in a higher rate of RTW (22).

To date, there is a lack of literature regarding whether and to what extent professional reintegration leads to a return to the same profession rather than a job change.

In the study by Weber et al., which prospectively examined 42 patients with acetabular fractures and an ISS 16–30 between 1988 and 1997. Hence the patient collective is comparable to the “polytrauma” subgroup in the present study. In line with our results, 74% of the patients had returned to work after a follow-up period of 2 years (4).

It should be emphasized that although vocational reintegration was addressed, the lack of detailed information regarding the program’s structure, duration, and quality limits the ability to fully evaluate their potential effectiveness.

### Workload according to the REFA-classification

A variety of studies focus on the post-traumatic change in work severity according to the REFA classification (18, 23–25). However, none of these studies refer to acetabular fractures. Accordingly, there is a lack of data on the occupation profile and corresponding work activities and work severity according to the REFA classification in patients suffering from an acetabular fracture.

The present study clearly shows that of the total collective, 6/22 (27.3%) had to reduce their workload POST according to the REFA classification. The median workload according to the REFA classification descriptively decreased by 50% after an acetabular fracture and more sedentary activities were performed (+25.6%). Aprato et al. investigated the RTW outcomes in a cohort over 100 patients who underwent surgical treatment for acetabular fractures. They found no significant association between RTW and sedentary occupations, but a significant correlation between return to the PRE occupation and sedentary occupations. They also found that sedentary occupations were associated with a 27.7% reduction in the duration of sick leave (5). In both subgroups, the percentage of individuals who were not employed POST increased by more than 20%. In the “polytrauma” subgroup, the median workload descriptively decreased to a greater (–50%) extent compared to the patients without polytrauma (–33.3%). Furthermore, the proportion of sedentary activities demonstrated a higher increase in the “polytrauma” subgroup (+40%) relative to the “no polytrauma” subgroup (+11.4%). In the “polytrauma” subgroup, the maximum POST REFA grade achieved was one level lower (REFA grade 2) compared to the “no polytrauma” subgroup (REFA grade 3), while the PRE grade was similar (REFA grade 4).

Interestingly, there is an inconsistency in the current literature on the association of RTW and ISS. Whereas some authors did not identify a significant relationship between the ISS and RTW outcome (6, 26), Gabbe et al., in contrast, demonstrated that patients with pelvic ring fractures and higher ISS had a significantly lower risk-adjusted probability of returning to work (27). Clay et al. (28) emphasized that the plentitude of variables used in the current literature may lead to inconsistent data and inconclusive results. However, the authors identified the ISS as a prognostic factor of RTW with only moderate evidence. Hence, future prospective clinical studies need to elucidate the association between RTW and ISS in orthopaedic trauma patients.

### Gender differences

In the total cohort, female participants showed a slightly higher RTW rate (80%) compared to males (73.33%), despite a lower baseline employment rate. However, males were more likely to reduce their workload post-injury, with over half reporting a decrease, while workload reduction among females was rare.

In the non-polytrauma subgroup, both males and the single female participant returned to work at high rates. Notably, male participants showed a substantial workload reduction, whereas the female participant maintained her pre-injury workload level.

In the polytrauma subgroup, the RTW rates were comparable in both sexes (75%). However, male participants more often reported a reduction in workload, while most female participants maintained their workload.

These findings suggest potential sex-specific differences in the RTW and possible workload. However, according to Aprato et al., there was no significant correlation between sex and post-traumatic job changes (5). Similarly, Ng et al. found no statistically significant association between sex and RTW or return to the same occupation as PRE (6). This may indicate that while workload adaptation differs, overall RTW likelihood is not strongly influenced by sex. Nevertheless, our findings underline the need for tailored, sex-specific rehabilitation approaches to optimize post-traumatic reintegration. The gender differences in workload can be found in Table IV

**Table IV:** Gender differences in workload according to the REFA classification

Group	*n*	Working PRE (*n*, %)	RTW rate (%, *n*)	Workload PRE (MD ± SD)	Workload POST (MD ± SD)	Descriptive median workload reduction (%)	Median individual workload reduction
Total – *Male*	15	15 (100)	73.33 (11/15)	2 ± 1.13	1 ± 0.67	50	1
Total – *Female*	7	5 (71.43)	80.00 (4/5)	2 ± 0.80	1.5 ± 0.58	25	0
No Polytrauma – *Male*	7	7 (100)	71.43 (5/7)	2 ± 1.15	1 ± 0.89	50	1
No Polytrauma – *Female*	1	1 (100)	100.00 (1/1)	1 ± 0	1 ± 0	0	0
Polytrauma – *Male*	8	8 (100)	75.00 (6/8)	1.5 ± 1.20	1 ± 0.41	33.33	0.5
Polytrauma – *Female*	6	4 (66.67)	75.00 (3/4)	2 ± 0.50	2 ± 0.58	0	0

REFA: Reichsausschluss für Arbeitszeitermittlung; *n*: sample size; PRE: pre-traumatic; POST: post-traumatic; MD: median; SD: standard deviation; RTW: return to work.

### Limitation

The limitations of this study are largely due to the small sample size (*n* = 22)., which restricts the statistical power needed for meaningful comparisons and subgroup analyses, such as those based on gender and trauma severity. These subgroup analyses are therefore susceptible to random variation, limiting the reliability of such findings. We emphasized the descriptive nature of our data.

Selection bias may also affect the study, as postal addresses were available for only 56 of 149 eligible patients with acetabular fractures, based on data from the administrative centre of the Level 1 Trauma Center. Of these, 15 could not be reached, and one was decreased, leaving 40 individuals who received the questionnaire. Ultimately, 22 completed responses were obtained. It is possible that respondents represent those with more favourable recovery or greater motivation, potentially overestimating return-to-work rates and underestimation of long-term impairments. In addition, subjects with acetabular fractures and concomitant pelvic ring fractures were also included, which means that the data cannot be explained in isolation by the effects of acetabular fractures alone. Furthermore, no conclusion can be drawn as to whether the changes in resilience at work and the existence of polytrauma are causally related to the presence of the acetabular fracture. The lack of a control group restricts the retrospective study design, and the long follow-up period (> 10 years) leading to a risk of recall bias and limit causal inferences. Whether and to what extent the self-reported answers to the questionnaires are objectively correct or influenced by other factors cannot be verified and lack objective validation. Further research including health insurance and employment records is needed.

Due to the descriptive character of the present study, we could not account for additional environmental factors (e.g. age, change of residence, change of friends, etc.), which could have influenced the occupation independently from the trauma.

Overall, further prospective studies are warranted to corroborate these preliminary observations.

### Conclusion

Possible workload was reduced after acetabular fracture. In the “polytrauma” as well as in the “no polytrauma” subgroup, the same percentage of participants returned to work. However, the presence of a polytrauma led to a return to an occupation with lower workload compared to the cases without polytrauma. Hence, polytraumatized patients with acetabular fractures require an intensified and prolonged rehabilitation process to prevent loss of workforce.
